# A High-Speed Imaging Method Based on Compressive Sensing for Sound Extraction Using a Low-Speed Camera

**DOI:** 10.3390/s18051524

**Published:** 2018-05-11

**Authors:** Ge Zhu, Xu-Ri Yao, Zhi-Bin Sun, Peng Qiu, Chao Wang, Guang-Jie Zhai, Qing Zhao

**Affiliations:** 1 Center for Quantum Technology Research, School of Physics, Beijing Institute of Technology, Beijing 100081, China; 2120111573@bit.edu.cn; 2 Key Laboratory of Electronics and Information Technology for Space Systems, National Space Science Center, Chinese Academy of Sciences, Beijing 100190, China; zbsun@nssc.ac.cn (Z.-B.S.); 09222016@bjtu.edu.cn (P.Q.); wangchao@nssc.ac.cn (C.W.); gjzhai@nssc.ac.cn (G.-J.Z.); 3 University of Chinese Academy of Sciences, Beijing 100049, China

**Keywords:** sound recovery, high-speed imaging, compressive sensing, computational imaging, remote sensing, vibration analysis

## Abstract

This paper reports an efficient method for sound extraction from high-speed light spot videos reconstructed from the coded light spot images captured with a low-speed camera based on compressive sensing, but at the expense of consuming time. The proposed method first gets the high-speed video of the light spot that is illuminated on the vibrating target caused by sound. Then the centroid of the light spot is used to recover the sound. Simulations of the proposed method are carried out and experimental results are demonstrated. The results show that high-speed videos with a frame rate of 2000 Hz can be reconstructed with a low-speed (100 Hz) charge-coupled device (CCD) camera, which is randomly modulated by a digital micro-mirror device (DMD) 20 times during each exposure time. This means a speed improvement of 20 times is achieved. The effects of synchronization between CCD image recording and DMD modulation, the optimal sampling patterns of DMD, and sound vibration amplitudes on the performance of the proposed method are evaluated. Using this compressive camera, speech (counting from one to four in Chinese) was recovered well. This has been confirmed by directly listening to the recovered sound, and the intelligibility value (0–1) that evaluated the similarity between them was 0.8185. Although we use this compressive camera for sound detection, we expect it to be useful in applications related to vibration and motion.

## 1. Introduction

The principle for sound detection is simple: sound waves hit the objects in their traveling path causing the objects to vibrate, and sensors detect these vibrations, resulting in useful information that can be used for sound recovery. Sound detection by optical means has become increasingly attractive due to its simple optical setup, the fact that it is non-destructive, and it is widely varied and used in important applications. These applications include surveillance in hostile environments, intrusion detection, abnormal situation detection in public places, and search and rescue [1,2]. Traditionally, non-contact optical devices such as Laser Doppler Vibrometers (LDVs) were used for sound recovery [3,4,5,6,7,8]. Generally, LDVs are based on the principle of laser interferometry, making LDVs highly sensitive to object surface reflections, environmental factors, and the mutual locations of the projection laser and the detection interferometer modules [9]. Recently, an emerging technology, image-based sound recovery from high-speed videos, has drawn much attention [10,11,12,13,14,15,16,17]. In these systems, a highly developed phase-based algorithm is applied to extract sounds from the high-speed videos that can show subtle motions [11]. Wang et al. [12] demonstrated a simpler and faster technique that can extract sounds from high-speed videos using a digital image correlation method. To further reduce the complexity of the computation, the authors in Refs. [13,14] use an efficient singular value decomposition (SVD)-based approach to recover sound information in the high-speed videos. In addition, it has been shown that with an appropriate optical schematic, the sound can be retrieved from the displacements [15] or the intensity variations [16,17] of the speckle patterns captured with a high-speed camera. Due to the high frame rates, high-speed cameras can record object motions, including sound vibrations, with less influence of circumstances. In addition, the high-speed camera records the entire area within its field angle. These systems allow one to separate sounds simply and easily [10]. However, traditional high-speed cameras need highly specialized and very expensive hardware [18] and they have to compromise spatial resolution in pursuit of a high frame rate. For example, the high-speed camera Phantom v710 of Vision Research can provide a spatial resolution of 1280 × 800 at a frame rate of 7530, but the spatial resolution has to be reduced to 128 × 128 when operating at a frame rate of 215,600 [19]. This trade-off between frame rate and spatial resolution is caused by the huge memory bandwidth required by high-speed videos. In addition, the video recording length is usually limited by the camera storage capacity. In References [20,21], the authors introduced an imaging modality that, by offsetting pixel-exposure time during the capture of a single image frame, embeds temporal information in each frame. This allows acquisition of high-speed sequences, but the spatial resolution is reduced.

Recent advances in computational imaging and compressive sensing [22,23,24,25,26] have enabled compressive cameras to capture fast phenomena at frame rates higher than that of the camera sensor [27,28,29,30,31,32,33,34,35,36,37]. Cameras designed based on these principles usually first encode the incoming video with a spatiotemporal light modulator according to pre-determined sampling patterns at a rate higher than the acquisition rate of the camera. That is, in each exposure time the incoming light is modulated several times. Thus, each captured frame is a multiplexed version of the original high-speed video voxel. Generally, the spatiotemporal light modulation is achieved either via a programmable pixel-wise spatial light modulator, such as a digital micromirror device (DMD) array [27], a liquid crystal on silicon (LCoS) mirror [28,29,30], or a printed coded-aperture mask placed on a fast-moving translation stage [31,32]. In addition, compressive cameras based on the flutter shutter modulation have also been devised [33,34]. As a second step, sparse reconstruction algorithms based on compressive sensing are used to reconstruct the high-speed video with high fidelity. The algorithms usually exploit the sparsity of the videos in the appropriate transform domains [31,32,33] or an over-complete dictionary [29,30,35], as well as the temporal redundancy in the videos [27,28].

In this paper, we propose a compressive camera imaging system to extract the sound from the reconstructed high-speed video of a light spot that is scattered from the vibrating object, which was caused to vibrate by the sound. A halogen lamp was collimated to a light spot and was employed to illuminate the target. A high frame-rate spatial light modulator of DMD was used to modulate the incoming light spot video during each exposure time, and a general low-speed CCD camera was used to record the modulated images. Then, the high-speed light spot video was reconstructed via algorithms based on compressive sensing. Finally, the centroid of the light spot was utilized to recover the sound. Simulations of the proposed technique were carried out and we report the experimental results here. To the best of our knowledge, there are no studies on sound recovery using a compressive camera.

The rest of this paper is organized as follows: firstly, the methods of sound recovery using our DMD compressive camera are introduced in Section 2. Then, the performance of the proposed method for sound recovery is investigated through simulations on the high-speed videos captured with a high-speed camera in Section 3. The experimental results are given in Section 4, where the effects of the synchronization between the CCD image recording and the DMD modulation, the optimal DMD sampling patterns, and the sound vibration amplitudes on the performance of the proposed method are described in detail. Moreover, the effectiveness of the proposed method has been verified by successfully extracting a speech that counting from one to four in Chinese. The discussions are given in Section 5. Finally, the conclusions are summarized in Section 6.

## 2. Methods

**Figure 1:**
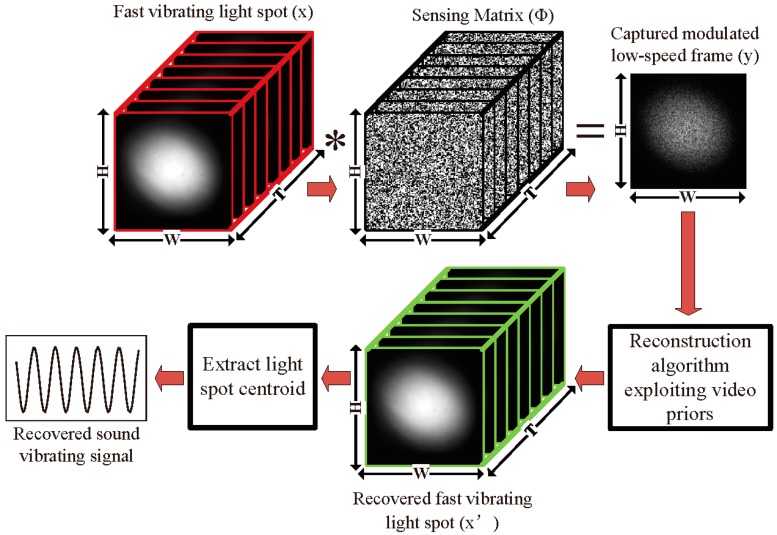
The schematic diagram of a compressive camera for sound detection. The forward sensing model includes a light spot video (x) consisting of T frames, with H×W pixels each, which is multiplied by T sampling patterns embedded within the sensing matrix Φ. The CCD (charge-coupled device) camera integrates over the exposure time, producing a single modulated image y consisting of H×W pixels. In the backward model, the algorithms based on compressive sensing are used to reconstruct the fast light spot video from the modulated images. Finally, the centroids of the reconstructed light spots are used to recover the sound.

The schematic diagram of the sound detection employing a compressive camera system is shown in Figure 1. The proposed method can be summarized in three key steps. First, the forward model maps a spatiotemporal volume of the vibrating light spot x onto a single image using the linear projective model (see Figure 1):

(1) y=Φx

where $\Phi \in R^{\left(H\cdot W\right)\times \left(H\cdot W\cdot T\right)}$ is a time-varying spatial sensing matrix that codes each of the T temporal channels of x prior to integrating them into one coded image $y\in R^{\left(H\cdot W\right)\times 1}$, and $x\in R^{\left(H\cdot W\cdot T\right)\times 1}$ is the unknown spatiotemporal volume that we want to reconstruct. Here, the elements of the matrix Φ are determined by a DMD with T random patterns. Let the patterns be denoted by the matrices $M_{i}\in R^{\left(H\times W\right)}$, i∈1,⋯,T. These are then used to populate a new matrix $M_{i}^{\prime }=diag\left(M_{i}\right)\in R^{\left(H\cdot W\right)\times \left(H\cdot W\right)}$, where function $diag\left(M_{i}\right)$ creates an (H·W)×(H·W) diagonal matrix with the elements of the vector $M_{i}$ on the main diagonal. The sensing matrix can then be expressed as a concatenation of these new matrices as

(2) $$\Phi =\left[M_{1}^{\prime }\;M_{2}^{\prime }\;M_{3}^{\prime }\;\cdots \;M_{T}^{\prime }\right].$$

Here, each of the patterns $M_{i}$ are chosen independently by programming the DMD with random patterns.

In the second step, we build a sparse reconstruction of the fast light spot video from the captured modulated low-speed images with algorithms based on compressive sensing. According to compressive sensing, inverting the under-determined system in Equation (1) for x requires additional assumptions. Modern reconstruction approaches such as dictionary-learning, maximum likelihood, and Bayesian algorithms, are able to decompress the data with varying degrees of success based on the spatial-temporal structure of the video. Of these, we use two iterative reconstruction algorithms called the Generalized Alternating Projection (GAP) [36] and the Two-step Iterative-Shrinkage Thresholding (TwIST) [37]. They exploit image and video priors to effectively solve the ill-posed inverse problem. The GAP utilizes the structural sparsity of the discrete frames in transform domains such as discrete cosine transform (DCT) or wavelets:

(3) $$\hat{a}=argmin_{a}{\left\|y-{\Phi \Psi }^{-1}a\right\|}^{2}+\lambda {\left\|a\right\|}_{1},$$

where Ψ represents the transform to the chosen domain resulting in a sparse representation a. Therefore, x is given by $x=\Psi^{-1}a$, and λ is an adjustable regularization parameter [36]. The GAP has a fast convergence time and it is based on the video’s global sparsity. Therefore, it is a universal reconstruction algorithm insensitive to the data being inverted [36]. In the following experiment, the DCT basis was used as the sparsifying basis as it was suitable for dynamic scene reconstruction, and λ was set to 1 for optimal performance, while TwIST employs a regularization function to penalize estimates of x that are unlikely or undesirable in the estimated $x_{e}$:

(4) $$x_{e}=argmin_{x}{\left\|y-\Phi x\right\|}^{2}+\lambda \Omega \left(x\right),$$

where Ω(x) and λ are the regularizer and regularization weights, respectively [37]. For the reconstruction in the simulations, a Total Variation (TV) regularizer was used:

(5) $$\Omega \left(x\right)=\overset{T}{\underset{k=1}{\sum }}\overset{W}{\underset{i=1}{\sum }}\overset{H}{\underset{j=1}{\sum }}\sqrt{{\left(x_{i+1,j,k}-x_{i,j,k}\right)}^{2}+{\left(x_{i,j+1,k}-x_{i,j,k}\right)}^{2}},$$

where k represents the *k*th temporal channel of x which contains T channels (see Figure 1). Several regularization weights were tested and a weight of λ=0.04 yielded the best performance and was used in the following simulations. The TV regularizer was chosen because many natural scenes are well-described by sparse gradients. In order to reconstruct the sub-frames from the coded frames using the compressive sensing algorithms, the measurement patterns of the DMD and the sparsifying basis should satisfy the incoherence condition. It has been demonstrated that random patterns yield a good incoherence with almost any sparsifying basis [22,23,24,25], and random patterns are easily implemented with DMD. Therefore, we selected random patterns. However, other measurement patterns and sparsifying bases may be better than the random patterns.

Third, we recover the sound vibration information from the reconstructed light spot images by tracking the centroid coordinates of each image, (see Reference [10]). Let $C_{x}$ and $C_{y}$ denote the centroid of a given image in the horizontal and vertical direction respectively. Then,

(6) $$C_{x}=\frac{\sum_{i=1}^{p}\sum_{j=1}^{q}i\cdot m\left(i,j\right)}{\sum_{i=1}^{p}\sum_{j=1}^{q}m\left(i,j\right)},C_{y}=\frac{\sum_{i=1}^{p}\sum_{j=1}^{q}j\cdot m\left(i,j\right)}{\sum_{i=1}^{p}\sum_{j=1}^{q}m\left(i,j\right)},$$

where m(i,j) is the intensity value of the pixel located at (i,j), and *p* and *q* indicate the row and column number of the image, respectively. It is reasonable to assume that the vibration amplitude of the light spot is proportional to the amplitude of the sound waves. Consequently, the amplitude of the sound wave at a sample point can be approximately reconstructed as

(7) $$C=\sqrt{C_{x}^{2}+C_{y}^{2}}.$$

## 3. Simulations

### 3.1. Simulation Setup

**Figure 2:**
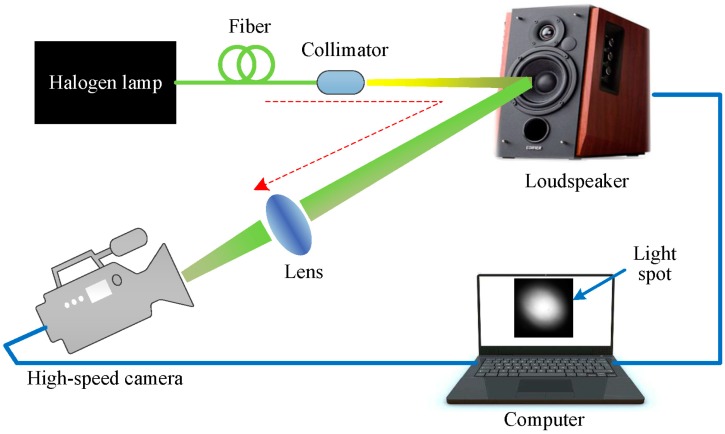
The simulation setup. The red dashed arrow line indicates the light path.

We first evaluate the performance of the proposed method for sound detection through noiseless simulations on sound vibrating light spot videos acquired using the simulation setup, as shown in Figure 2. For simplicity, a loudspeaker was used as the sound source. The output light of a fiber-coupled halogen lamp was collimated and incident on the membrane of the loudspeaker. The scattered light was focused into a light spot via a lens and recorded by a high-speed camera (Phantom v7.3, Vision Research) at a frame rate of 2000 fps (frames per second).

### 3.2. Simulation Results

**Figure 3:**
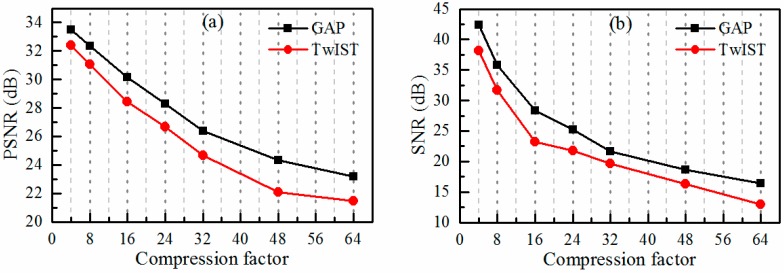
The simulation results for a sinusoidal audio signal with a frequency of 50 Hz. (**a**) The reconstruction quality of the light spot video images as a function of the compression rate for both the GAP (Generalized Alternating Projection) and TwIST (Two-step Iterative-Shrinkage Thresholding) algorithms; (**b**) the quality of the sound signal recovered by the reconstructed sound vibrating light spot video as a function of the compression rate.

In the first simulation, we investigated the effect of the compression rate (varying T in Figure 1) on the performance of the video reconstruction using the two reconstruction algorithms mentioned above. We also evaluated the quality of the sound recovery. The sound vibrating light spot video was recorded while the loudspeaker emitted a sinusoidal sound with a frequency of 50 Hz (hertz). With regard to each algorithm, we used random coding matrices. The simulated observations were obtained by sampling the high-speed video using the linear projective model of Equation (1). Figure 3a shows the reconstruction quality of the light spot image for each algorithm in terms of the average peak signal-to-noise ratio (PSNR). As the compression rate increases, the reconstruction quality of both the two algorithms decay, with the GAP algorithm consistently approximately 2 dB better than the TwIST algorithm. Meanwhile, the signal-to-noise ratio (SNR) of the recovered sound signal is shown in Figure 3b. The SNRs decrease with the increase in the compression rate for both of the algorithms, with the GAP algorithm performing consistently better than the TwIST algorithm. It also shows that the SNRs decrease quicker when the compression rate increases from 4 to 16 than when the compression rate increases from 16 to 64.

**Figure 4:**
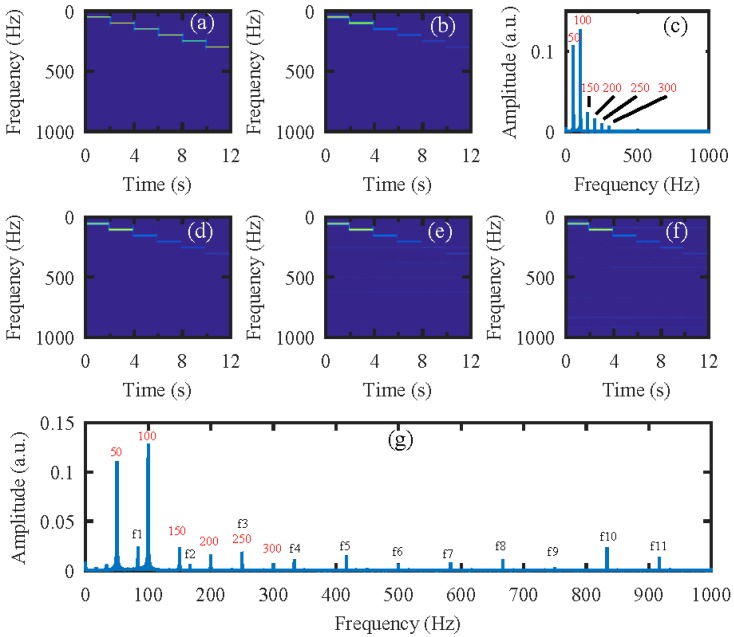
The simulation results for an audio signal with increasing frequencies from 50 Hz to 300 Hz with an interval of 50 Hz. Each segment lasts 2 s. (**a**) The original audio spectrogram. (**b**) The spectrogram of audio reconstructed directly from the high-speed video and (**c**) its frequency spectrum. The spectrogram of audio reconstructed from the high-speed video reconstructed using the compressive camera with (**d**) 8×, (**e**) 16×, and (**f**) 24× compression. (**g**) The frequency spectrum of the signal in (**f**). The red text of 50 to 300 Hz in (**c**) and (**g**) indicate the expected signal, while f1 to f11 in (**g**) indicate the undesired harmonic noise related to the frame rate (2000/24 Hz) of the compressive camera.

Next, the simulation was performed on the video of the vibrating light spot recorded while the loudspeaker played a set of sinusoidal sound segments with increasing frequencies ranging from 50 Hz to 300 Hz with an interval of 50 Hz. Each segment lasted 2 s, as shown in the audio spectrogram in Figure 4a. Figure 4b is the audio spectrogram reconstructed directly from the high-speed video with the centroid method. Figure 4c shows the frequency spectrum of the signal in Figure 4b. Figure 4d–f present the audio spectrograms reconstructed from the high-speed video reconstructed with the compressive camera with 8×, 16×, and 24× compression, respectively. In this simulation, the GAP algorithm was used. In Figure 4d through to Figure 4f, we see that when the compression rate is small, the reconstructed audio spectrogram (see Figure 4d) is very similar to the original one. However, for a larger compression rate, the noise is more apparent (see Figure 4e,f). Figure 4g shows the frequency spectrum of the signal in Figure 4f; we can see from these figures that, except for the expected signal (the red text of 50 to 300 Hz in Figure 4c,g), there is also signal from the undesired noise (indicated by f1–f11 in Figure 4g). Moreover, the frequencies of f1 through f11 indicate that they are harmonics of the frame rate of the compressive camera (2000/24 Hz). Since the frame rate is known, the harmonic noise can be suppressed easily.

**Figure 5:**
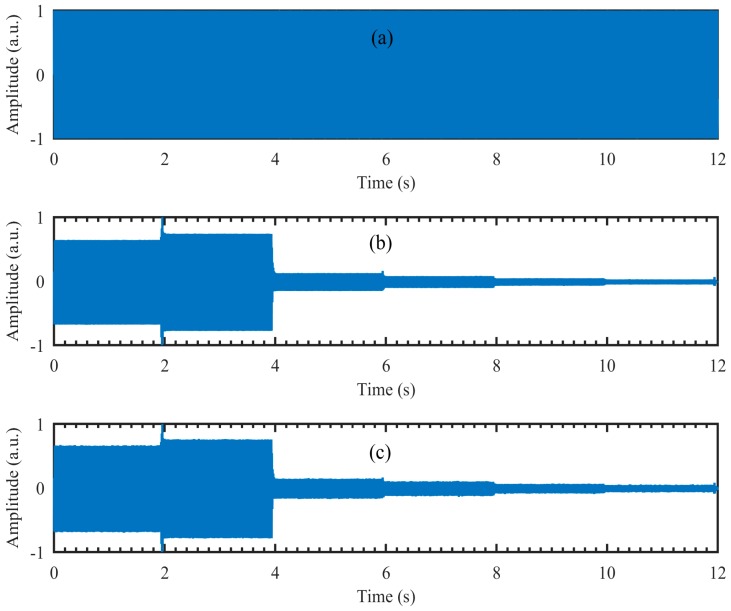
(**a**) The original audio waveform; (**b**) the audio waveform recovered directly from the high-speed video captured by the high-speed camera; (**c**) the audio waveform recovered from the reconstructed high-speed video using the compressive camera with 8× compression.

From Figure 4c,g we can see that the amplitude of the recovered segmented audio at low frequencies is larger than that at high frequencies. To manifest the possible reasons, the audio waveforms are given in Figure 5. The audio waveforms in Figure 5a–c correspond to the audio spectrogram in Figure 4a,b,d, respectively. Figure 5a is the original audio waveform and the amplitude of each segment audio is equal. Figure 5b is the audio waveform recovered directly from the high-speed video captured by the high-speed camera, while Figure 5c is the audio waveform recovered from the reconstructed high-speed video using the compressive camera with 8× compression. It can be seen that the audio waveforms in Figure 5b,c are almost the same and compared with Figure 5a, their amplitudes at low frequencies are larger than those at high frequencies. The reason may be due to the stronger response of the loudspeaker membrane to the low-frequency sound waves than to the high-frequency ones.

**Figure 6:**
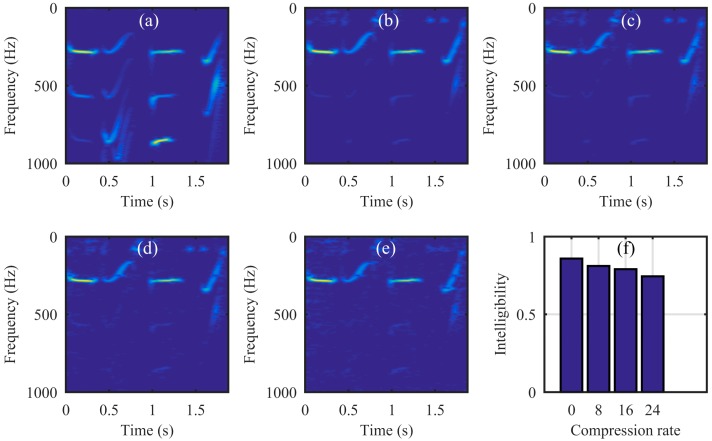
The simulation results for complex sound (speech containing counting from one to four in Chinese). (**a**) The original audio (see supplementary, Media1) spectrogram; (**b**) the audio (see supplementary, Media 2) spectrogram reconstructed directly from the high-speed video, reconstructed audio spectrogram from the high-speed video reconstructed using the compressive camera with 8× (**c**) (see supplementary, Media 3), 16× (**d**) (see supplementary, Media 4) and 24× (**e**) (see supplementary, Media 5) compression and (**f**) intelligibility values (0–1) that evaluate the similarity between the reconstructed sounds and the test one.

**Figure 7:**
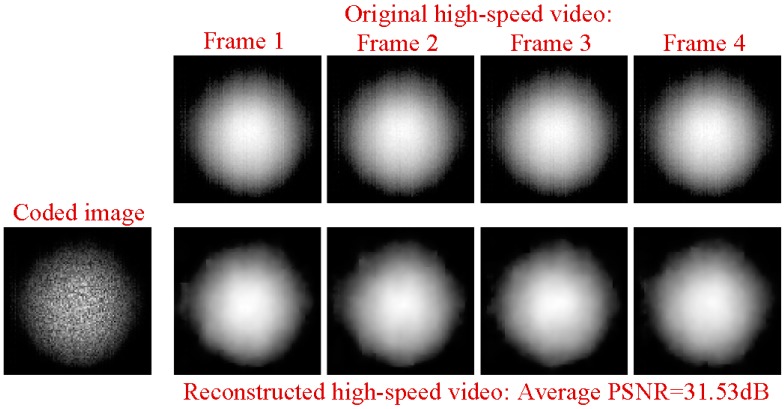
The comparison of the reconstructed high-speed video with the original one at the case of 16× compression. (**Top row**): the first four frames of the original high-speed video. (**Bottom row**): the corresponding reconstructed video and the coded image. The high-speed video in the bottom row are reconstructed from the coded image.

Next, we ran a simulation with more complex sounds: speech containing counting from one to four in Chinese. There are two main reasons that why we select counting one to four in Chinese as a test signal. One is that the test signal lasts just 1.876 s, making the computation time not too much whilst still having enough information to estimate the performance of our proposed method. The more important reason is that the frequency components of the test signal concentrate at 0 Hz to 1000 Hz, while the maximum frame rate of the realized compressive camera is 2000 Hz, which is determined by the maximum modulation rate of the DMD. Thus, according to Nyquist’s Theorem, the test signal can be measured using the compressive camera. Therefore, other sounds with different frequency contents can be selected as long as their frequencies are two times less than the frame rate of the compressive camera. Figure 6a shows the original audio (see supplementary, Media 1) spectrogram played by the loudspeaker. Figure 6b shows the reconstructed audio (see supplementary, Media 2) spectrogram obtained directly from the high-speed video. Figure 6c (see supplementary, Media 3), Figure 6d (see supplementary, Media 4), and Figure 6e (see supplementary, Media 5) show the spectrogram from audio reconstructed from the high-speed video reconstructed using the compressive camera with 8×, 16×, and 24× compression, respectively. The corresponding average PNSRs that evaluated the reconstruction quality were 32.50 dB, 31.38 dB, and 30.70 dB, respectively. Since the frame rate of the compressive camera was known, in Figure 6c–e, the noise related to the camera frame rate was suppressed by digital filtering. The reconstructed sounds match the original one quite well. This finding has been confirmed by directly listening to the test sound and the reconstructed ones. A quantitative evaluation of the similarity between the reconstructed and test sounds is given in Figure 6f. The values of the intelligibility [38] (0–1) between the test sound and the reconstructed ones in Figure 6b–e were 0.8590, 0.8114, 0.7907, and 0.7448, respectively. To further demonstrate the reconstruction performance of the high-speed light spot video, Figure 7 presents the comparison of the reconstructed high-speed video with the original one in the case of 16× compression. The top row in Figure 7 is the first four frames of the original high-speed video, while the bottom row is the corresponding reconstructed frames and the coded image. The high-speed video in the bottom row is reconstructed from the coded image and the average PSNR of the reconstruction was 31.53 dB.

## 4. Experiments 

### 4.1. Experimental Setup

**Figure 8:**
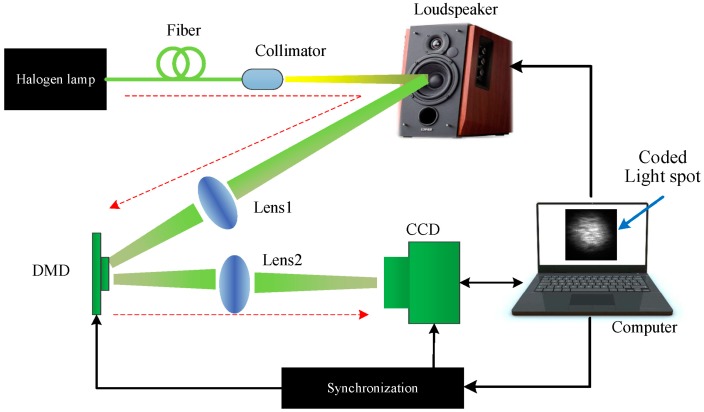
The experimental setup. The red dashed arrow line indicates the light path.

Finally, an experiment was conducted using the setup in Figure 8. The experimental setup consisted of a halogen lamp, the light of which was collimated and then illuminated the test target (a loudspeaker); a DMD (1024 × 768 micromirrors, 13.68 μm^2^/mirror) [39] to perform coded exposure, with a modulation that can be controlled by external trigger and a maximum modulation rate of 2000 Hz; two imaging lenses (lens1 and lens2, Nikon enlargement lens, f = 50 mm); a CCD camera (Manta G-145-30fps, 1388 × 1038 pixels, 6.45 μm^2^/pixel) [40] to record the coded images, and with image recording that can be triggered by an external clock signal, and at full resolution a maximum frame rate if 30.1 frames per second; a synchronization module to synchronously trigger the DMD modulation and CCD image recording, and a delay between them that can be adjusted; and a computer to generate the random sampling patterns for the DMD modulation, to perform the reconstructions of the vibrating light spot video from the coded images and to recover the sound from the reconstructed video. Additionally, to improve the intrinsic speed of the CCD camera itself, the active region of the CCD sensor that we selected was 96 × 96 pixels. Thus, the highest speed of the CCD was 110 Hz. However, when the CCD worked at the highest frame rate of 110 Hz, it seriously lost frames. In order to avoid losing frames, the frame rate of 100 Hz was selected. In addition, the distance between the compressive camera and the loudspeaker was about 80 cm.

**Figure 9:**
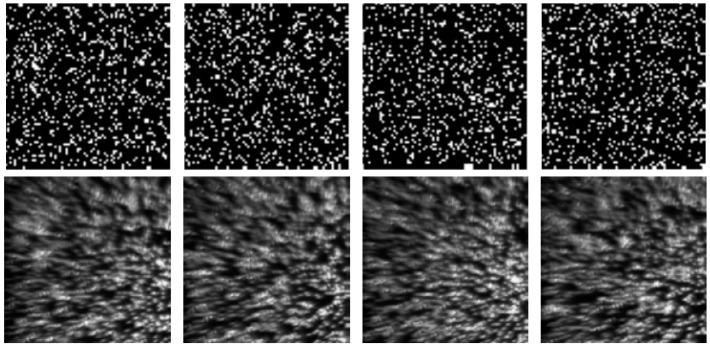
The comparison of the designed sampling patterns with the used image patterns.

For the optical system, the incident light from the halogen lamp was first scattered from the vibrating membrane of the loudspeaker, converged into one light spot and projected onto the DMD plane via Lens1. Then, the light spot was coded by the sampling pattern of the DMD. Each micro-mirror of the DMD is addressable. It can rotate about a hinge and can be shifted between two directions of +12° (1) or −12° (0) with respect to the surface of the DMD. Therefore, the light can be reflected in two directions depending on the pattern encoded on the DMD. In our experimental setup, we set the active reflected light to −12°, and the reflected light was directed to the CCD via Lens2. During each camera exposure time, the DMD synchronously displayed several random binary patterns corresponding to the sampling function. Therefore, the significant problem was how to make the correspondence pixel-to-pixel between the DMD mirrors and the CCD pixels with high accuracy. However, since the CCD and DMD have different pixel sizes, the one-to-one correspondence requires accurate geometric and photometric calibration, which makes the alignment difficult and time-consuming. To simplify the calibration procedure, a set of patterns $M_{i}$ were recorded with the CCD camera while the DMD, with the preset random sampling patterns, was illuminated by uniform light. The recorded image patterns $M_{i}$ were then used to populate the sensing matrix Φ using Equation (2). Figure 9 shows the comparison of four designed sampling patterns (the first row) with the corresponding recorded image patterns (the second row). Here, the ratio of the number of zeros to the ones in each designed sampling pattern was 15%. In these patterns, as shown in the first row of Figure 9, the zeros and ones were represented by white and black points, respectively. From Figure 9 we can see that the resulting Φ matrix was no longer identical to the designed one, which may introduce some deterioration into the experimental performance.

### 4.2. Experimental Results

**Figure 10:**
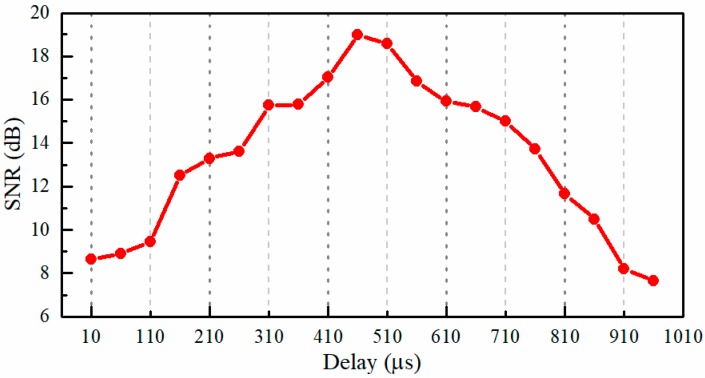
The dependence of the SNR (signal-to-noise ratio) of the recovered sinusoidal sound signal on the delay between the signals that trigger the CCD camera recording and DMD (digital micro-mirror device) modulation. The frequency of the sinusoidal voltage applied to the loudspeaker is 150 Hz, the rate of CCD image recording is 100 Hz, and the rate of DMD modulation is 800 Hz.

In the first experiment, we investigated the influence of the delay between the signals that triggered the CCD camera recording and the DMD modulation on the performance of the reconstruction. In this experiment, the loudspeaker membrane was used as the test object. The loudspeaker was driven by a sinusoidal voltage with a frequency of 150 Hz, which was supplied by a function generator of AFG3022C (Tektronix, Beaverton, OR, USA), and the peak-to-peak amplitude was 0.464 V. Although we do not know the loudspeaker’s characteristics and how well the loudspeaker transforms electric oscillations to the mechanical ones, when the sinusoidal voltage was applied to the loudspeaker, it gives reason to believe that the vibration of the loudspeaker membrane was also sinusoidal since the harmonic oscillations were the most elementary [40]. Therefore, an analysis of sound recovered quality can be performed by the SNR. In addition, the recording speed of the CCD camera was 100 fps, and in each exposure time, the incoming light was randomly modulated 8 times by the DMD. Thus, the light spot video with a frame rate of 800 Hz (8× compression) can be reconstructed. Figure 10 shows the dependence of the SNR of the recovered sinusoidal sound vibration on the delay between the triggering signals for the CCD camera recording and the DMD modulation. The delay was varied from 10 μs to 960 μs with a step of 50 μs. It can be seen that, at the delay of 460 μs, the highest SNR value of 18.98 dB was obtained. In the following experiments, the delay between the triggering signals for the CCD camera recording and the DMD modulation will be set to 460 μs.

**Figure 11:**
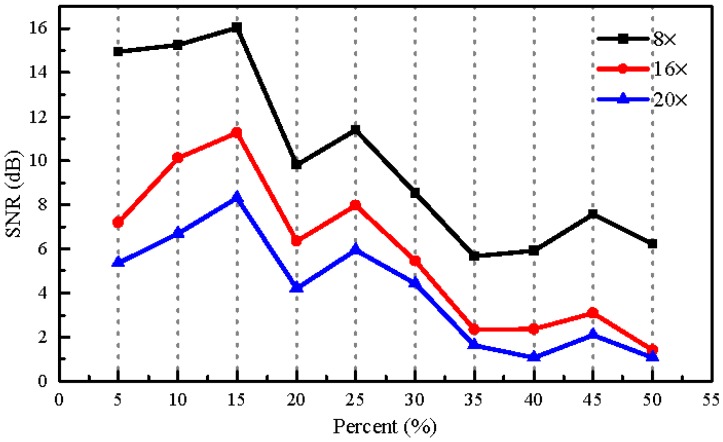
The dependence of the SNRs of the recovered sinusoidal sound signal on the ratio of the number of zeros to ones in each designed sampling pattern at different compressions of 8×, 16×, and 20×. The frequency of the sinusoidal voltage applied to the loudspeaker is 150 Hz and the rate of CCD image recording is 100 Hz.

In order to determine the optimal random sampling patterns for use in our experiments, we performed an experiment comparing the sound recovery performance in terms of SNR for different ratios of the number of zeros to the ones in each designed sampling pattern with different compressions of 8×, 16×, and 20×. Here, the reason why we chose the compression of 20× was that the fastest rate of the DMD we used is 2000 Hz. Therefore, for the CCD image recording rate of 100 Hz, the maximum compression was 20×. In this experiment, the frequency of the sinusoidal voltage applied to the loudspeaker was 150 Hz and its peak-to-peak amplitude was 0.464 V. The rate of the CCD image recording was set to 100 Hz. Figure 11 gives the results, from which it can be seen that with the ratio of 15% the optimal performance is obtained. The top row in Figure 9 shows the designed patterns for the DMD. The white points denote the zeros in the designed patterns while the black points denote ones, and the ratio between the numbers of zeros to ones was 15%. The bottom row in Figure 9 shows the corresponding imaged patterns used for reconstruction. Compared to the designed patterns, the overlap in the imaged patterns is obvious. Increasing the ratio between the numbers of zeros to ones in the designed patterns can increase the measured information, but the overlap between the image pixels increases as well. Thus, the optimal results with designed patterns of 15% may be the tradeoff between the measured information and the spatial resolution of the captured image. Moreover, as was expected, when the compression was increased the performance deteriorated accordingly. In the following experiments, the ratio of the number of zeros to the ones in each used sampling pattern will be set to 15%.

**Figure 12:**
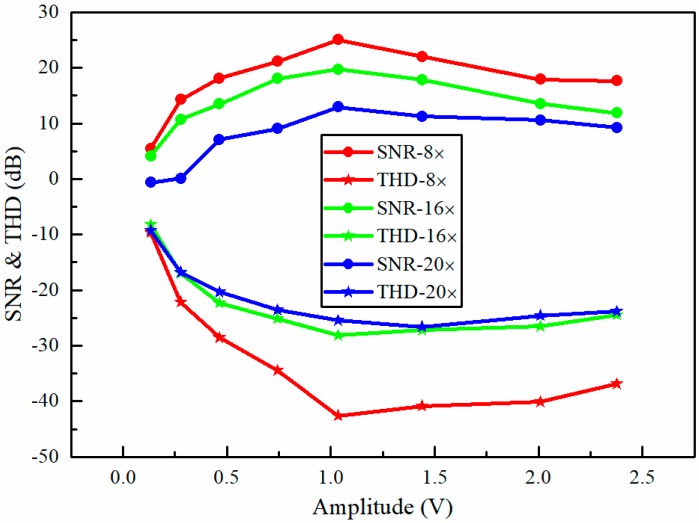
The dependence of the SNR (circle symbol) and THD (total harmonic distortion) (star symbol) of the recovered sinusoidal sound signal on the amplitude of the sinusoidal voltage applied to the loudspeaker at the different compressions of 8× (red points), 16× (green points), and 20× (blue points). The frequency of the sinusoidal voltage applied to the loudspeaker is 150 Hz and the rate of CCD image recording is 100 Hz.

Theoretically, the vibration amplitude of the light spots is a major factor that affects the performance of our proposed method. Here, it was investigated by comparing the SNR and total harmonic distortion (THD) of the reconstructed sound signals [41]. The membrane of the loudspeaker was still used as the test object. The loudspeaker was playing a sinusoidal sound with a constant frequency of 150 Hz. The vibration amplitudes were adjusted by changing the peak-to-peak amplitude of the sinusoidal voltage applied to the loudspeaker. Figure 12 shows the sinusoidal sound recovered quality (in terms of SNR and THD) for different sound vibration amplitudes at the different compressions of 8×, 16×, and 20×. The results demonstrate that the best recovered quality was obtained at a medium amplitude of 1.036 V. For large sound vibration amplitudes, the deterioration of the recovered quality may be caused by the larger harmonic distortions.

**Figure 13:**
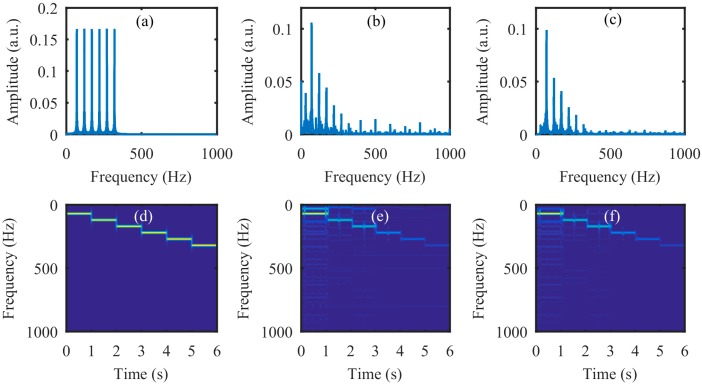
The experimental results for an audio signal consisting of six sinusoidal sound segments with increasing frequencies from 70 Hz to 320 Hz with an interval of 50 Hz. Each segment lasted one second. (**a**) The test sound frequency spectrum; (**b**) the recovered sound frequency spectrum without filtering and (**c**) with filtering; (**d**) the test audio spectrogram; (**e**) the recovered audio spectrogram without filtering and (**f**) with filtering.

To further test the validity of our proposed method for sound detection, we used a test sound consisting of six sinusoidal sound segments with increasing frequencies from 70 Hz to 320 Hz with an interval of 50 Hz. Each segment lasted one second, as shown by the audio spectrogram in Figure 13d; Figure 13a is the corresponding test sound frequency spectrums. In the experiment, the frame rate of the CCD was 100 Hz and in each exposure time, the incoming light was randomly modulated 20 times by the DMD. Thus, the light spot video with a frame rate of 2000 Hz can be reconstructed. This means a 20× speed improvement for the CCD camera was achieved. Figure 13b,c show the recovered sound frequency spectrum using the GAP algorithm without filtering and with filtering, respectively. Comparing Figure 13a,b we can see that the noise frequencies are mainly harmonics of 100 Hz (the frame rate of the CCD), which can be suppressed via simple digital filtering (see Figure 13c). Figure 13e,f show the recovered sound audio spectrogram without filtering and with filtering, respectively. From Figure 13, it is also seen that the tested loudspeaker membrane has a stronger response to low-frequency audio waves than to high-frequency ones.

**Figure 14:**
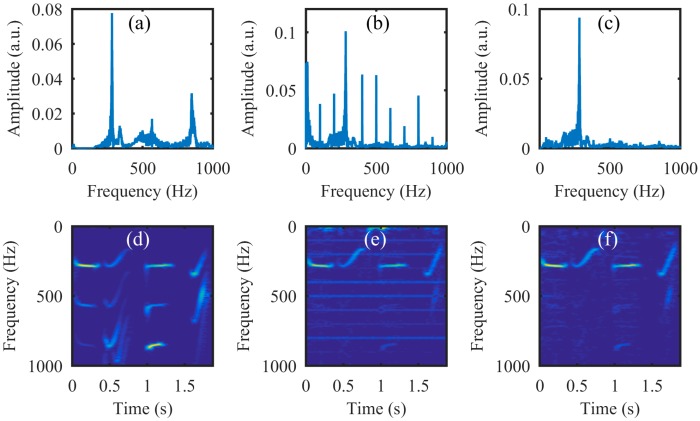
The experimental result for speech (counting from one to four in Chinese). (**a**) The test sound (see supplementary, Media 6) frequency spectrum; (**b**) the recovered sound frequency spectrum without filtering and (**c**) with filtering (see supplementary, Media 7); (**d**) the test audio spectrogram; (**e**) the recovered audio spectrogram without filtering and (**f**) with filtering.

**Figure 15:**
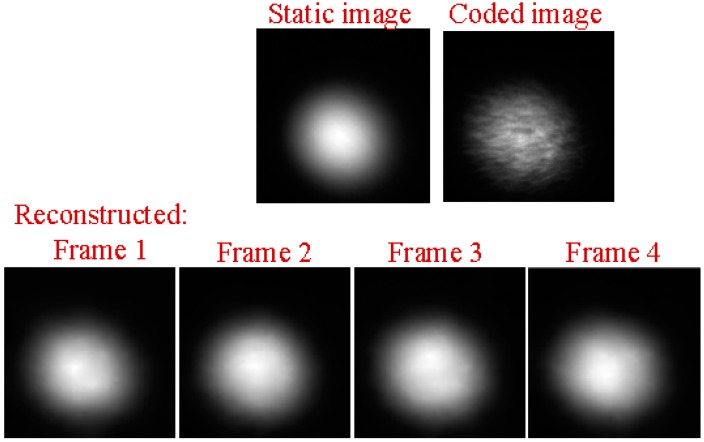
(**Top row**): the left is a static light spot image; the right is a coded image at the case of 20× compression for sound recovery. (**Bottom row**): the first four frames of the high-speed video reconstructed from the coded image in the (**Top row**).

In the next experiment, we tried to recover a speech signal, (counting one to four in Chinese) (see supplementary, Media 1). Figure 14a,d show the frequency spectrum and audio spectrograms of the test sound. To capture the sound, the CCD recording rate was still 100 Hz and the video compression was still 20×. This means that high-speed videos with a frame rate of 2000 Hz can be reconstructed. Figure 14b,e present the frequency spectrum and audio spectrogram of the recovered sound (see supplementary, Media 6) without any noise removing process. It is clear that the noise is mainly concentrated at the harmonics of the frame rate (100 Hz) of the CCD. Thus, clear sound (see supplementary, Media 7) can be extracted via digital filtering, as shown in Figure 14c,f. In addition, the intelligibility value, which is a measure of the similarity between the recovered sound without filtering and the test one, was 0.6317. After simple digital filtering, the intelligibility was improved to 0.8185. The digital filter was a digital Chebyshev Type I Stopband filter with multiple Stopbands, and their center frequencies were 100 Hz, 200 Hz, 300 Hz, 400 Hz, 500 Hz, 600 Hz, 700 Hz, 800 Hz, and 900 Hz, respectively. The left and right cutoff frequencies of the passband were [center frequency − 5 Hz] and [center frequency + 5 Hz]. The left and right cutoff frequencies of the stopband were [center frequency − 10 Hz] and [center frequency + 10 Hz]. In addition, the passband ripple and stopband attenuation were 0.1 dB and 0.4 dB, respectively. The digital filter was designed with the MATLAB functions of cheb1ord and cheby1, and the recovered sound signal was filtered with the MATLAB function, filter. Comparing Figure 14c,f with Figure 14a,d, it can be seen that the amplitudes of the recovered audio at low-frequencies are comparable with the test audio, while the amplitudes of the recovered audio at high-frequencies are much smaller than the test audio. This finding may be due to the stronger response of the tested loudspeaker membrane to the low-frequency sound waves than to high-frequency ones. Figure 15 shows the first four frames of the high-speed video reconstructed from the coded image in the case of 20× compression. Compared with the static light spot image on the left of the first row, we can see that the high-speed video images are reconstructed well.

## 5. Discussions

We have presented a prototype compressive video camera capable of 2000 fps reconstructions with a traditional low-speed CCD camera of 100 fps for sound extraction. To the best of our knowledge, this was the first time to use the compressive camera for sound detection. In our prototype, a spatial light modulator of DMD is employed. Compared with the modulator of LCoS, the advantage of using DMD is that no polarizer is needed and also that the reflectivity of DMD mirror is higher than that of LCoS, so the light throughput of DMD should be higher. However, since the modulation is achieved by tilting the micro-mirror, the DMD plane may be not parallel to the image sensor plane, thus, the lens aberration (see Figure 9) increases markedly [42]. In addition, we used the imaged sampling patterns as the sensing matrix, which was no longer identical to the designed sampling patterns, introducing some deterioration to the reconstructed quality of the high-speed video. We believe that with accurate geometric and photometric calibration and high-quality optic components, one-to-one correspondence between the CCD camera and DMD can be obtained. Thus, the performance of the method can be improved. Although we evaluated the performance of our compressive camera for sound extraction with temporal resolution increases of 8×, 16×, and 20×, a higher temporal resolution is probable. However, as demonstrated by the simulation and experimental results, by increasing the temporal resolution, the quality of the reconstructed high-speed video will be deteriorated.

The sound signal with frequency components that are not too high, such as 0 Hz to 1000 Hz, will benefit more from this approach. According to Nyquist’s Theorem, the maximum frame rate of the compressive camera should be two times larger than the highest frequency of the sound signal. However, the maximum frame rate of the compressive camera is determined by the maximum modulation rate of the spatial light modulator (SLM), such as the maximum frame rate of the DMD used in our paper (2000 Hz), and the higher the maximum modulation rate of the SLM, the higher the price. When the frame rate of the low-speed is fixed, to increase the speed of the compressive camera, the modulation rate of the SLM will be increased, as well as the number of the sub-frames reconstructed from one coded image. However, by increasing the number of the sub-frames that are reconstructed from one coded image, the quality of the reconstructed frames deteriorates and the computation time increases as well.

**Figure 16:**
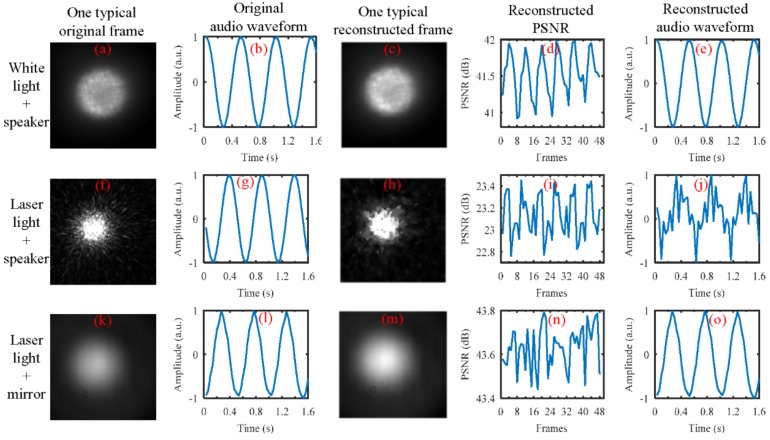
The comparison of the performance of the white light source and the laser light source for sound recovery. The sound vibration was generated by the loudspeaker, which was driven by a 2 Hz sinusoidal sound signal. The reflected light spot video was captured by the CCD camera with a frame rate of 30 Hz. (**Top row**): the white light was illuminated on the loudspeaker membrane. (**Middle row**): laser light was illuminated on the loudspeaker membrane. (**Bottom row**): laser light was illuminated on a reflecting mirror that was taped onto the loudspeaker membrane. From left to right, (**First column**): one typical frame captured by the CCD camera. (**Second column**): recovered audio waveform directly from the captured video by the CCD camera. (**Third column**): one typical reconstructed frame using the GAP algorithm, and eight frames were reconstructed from each coded frame. (**Fourth column**): the quality of the reconstruction in terms of PSNR. (**Fifth column**): the recovered audio waveform from the reconstructed video.

In our study, we found that the kind of the light source seems to have an important influence on our proposed method for sound recovery. From our simulations, the results demonstrated that whether we used laser light (λ=632.8 nm) spot videos or white light (halogen lamp light) spot videos, the high-speed videos can be well reconstructed from the coded low-speed videos with both of the reconstruction algorithms we considered (GAP and TwIST), as shown in Figure 16. In the simulation, the CCD camera with a frame rate of 30 Hz was used to capture the reflected light spot videos from the loudspeaker, which was driven by a 2 Hz sinusoidal sound signal. From the top row to bottom row, three cases were considered. The results in the top row were obtained when the white light was illuminated on the loudspeaker membrane. The results in the middle row were obtained when the laser light was illuminated on the loudspeaker membrane, while the results in the bottom row were obtained when the laser light was illuminated on a reflecting mirror that was taped on the loudspeaker membrane. From left to right, the first column shows one typical frame directly captured by the CCD camera. The second column shows the recovered audio waveforms directly from the captured video by the CCD camera. The third column shows one typical reconstructed frame using the GAP algorithm and the quality of the reconstruction in terms of PSNR is shown in the fourth column. The fifth column is the recovered audio waveforms from the reconstructed video. From the three cases, we can see that the audio waveforms can well be recovered from the videos directly captured by the CCD camera (see Figure 16b,g,f). However, only the reconstructed white light spot videos (see Figure 16d) and the reconstructed laser light spot videos obtained from the reflecting mirror (see Figure 16m) can be used to recover the sound correctly (see Figure 16e,o). In our opinion, this may be caused by the fact that the structure of the laser light spot is random speckle pattern generated when coherent light is illuminated on the rough surface of the loudspeaker’s membrane, and the structure of the random speckle pattern may vary with the vibration of the loudspeaker’s membrane. This deserves further investigation. Anyway, for practical applications based on active lighting, the laser light has advantages over the white light.

From the top row and bottom row of Figure 16, we can see that the captured frames are weak, but the method still works. Therefore, the light needed for the method to work is not too much. However, to quantify the influence of the light intensity on the performance of the method is significant, and will be studied in our future work. The working distance of the setup is another important performance indicator, especially for long-distance measurements. It is mainly determined by the emitting power of the light source, the reflectivity of the surface of the target, the attenuation of the atmosphere, as well as the transmission coefficient of the optic associated with the emitter and receiver. This will be further investigated in our future work.

In this paper, we primarily investigated the feasibility of using the high-speed video reconstructed from the compressive camera with a traditional low-speed camera for sound recovery. Although we illuminated the light on just one object, the camera system has the ability of spatial resolution. With the appropriate optics illuminating more than one object, the method can also extract different objects’ vibration from the reconstructed high-speed video. Although the algorithms of GAP and TwIST and random modulation patterns were used in our paper, as demonstrated in the paper of Reference [32], other modulation patterns and algorithms can also be used for measurement and reconstruction. With the tradeoff of the computation time and reconstruction quality, the GAP algorithm usually works better [32]. The sparsifying bases also have an important influence on the performance of the reconstructed video, such as the fact that the DCT basis is more suitable for the GAP algorithm for dynamic scenes reconstruction. Moreover, using learning dictionary trained with specific videos as the sparsifying basis may be able to improve the reconstruction quality when laser light is used as the light source. An in-depth analysis of the modulation patterns and sparsifying bases will be carried out in the near future.

## 6. Conclusions

In conclusion, we developed a high-speed imaging system with a general low-speed CCD camera based on compressive sensing for sound recovery. The incoming light spot video was modulated by a spatial light modulator of DMD before recording by the CCD camera. The feasibility of the proposed method for sound recovery has been demonstrated using detailed simulation and experimental studies. The effects of synchronization between CCD image recording and DMD modulation, the optimal sampling patterns of DMD, and the sound vibration amplitudes on the performance of the proposed method were evaluated. The results show that high-speed videos with a frame rate of 2000 Hz can be reconstructed with a low-speed (100 Hz) CCD camera, which is randomly modulated by the DMD 20 times during each exposure time. This means a speed improvement of 20 times is achieved. Using the high-speed (2000 Hz) compressive camera, we showed that a speech (counting one to four in Chinese) can be well recovered with the compressive GAP algorithm. This has been verified by directly listening to the test sound (see supplementary, Media 1) and the recovered one (see supplementary, Media 7). The intelligibility value (0–1) that evaluated the similarity between them was 0.8185. Through our experiments, we used the imaged sampling patterns for the reconstruction of the high-speed video. We believe that the pixel-to-pixel correspondence between the CCD camera and DMD can further improve the performance. Since high-speed sub-frames need to be reconstructed from the low-speed coded frames using the algorithms based on compressive sensing, the computation is time-consuming and faster algorithms are favorable. In addition, the influence of the light source on the performance of the proposed method deserves to be further investigated. In this paper, we used the developed compressive camera for sound detection, but it is also expected to be useful in many other applications related to vibration and motion.

## Supplementary Materials

The following are available online at http://www.mdpi.com/1424-8220/18/5/1524/s1.
